# Midkine: A Novel and Early Biomarker of Contrast-Induced Acute Kidney Injury in Patients Undergoing Percutaneous Coronary Interventions

**DOI:** 10.1155/2015/879509

**Published:** 2015-01-05

**Authors:** Jolanta Malyszko, Hanna Bachorzewska-Gajewska, Ewa Koc-Zorawska, Jacek S. Malyszko, Grazyna Kobus, Slawomir Dobrzycki

**Affiliations:** ^1^2nd Department of Nephrology and Hypertension with Dialysis Unit, Medical University, M. Sklodowskiej-Curie 24a, 15-276 Bialystok, Poland; ^2^Department of Invasive Cardiology, Medical University, M. Sklodowskiej-Curie 24A, 15-276 Bialystok, Poland; ^3^Department of Clinical Medicine, Medical University, Szpitalna 37, 15-254 Bialystok, Poland

## Abstract

We tested the hypothesis whether midkine could represent an early biomarker of contrast-induced acute kidney injury (CIAKI) in 89 patients with normal serum creatinine undergoing PCI. Midkine, serum and urinary NGAL, and cystatin C were evaluated before and 2, 4, 8, 24, and 48 hours after PCI using commercially available kits. Serum creatinine was assessed before and 24 and 48 hours after PCI. We found a significant rise in serum midkine as early as after 2 hours (*P* < 0.001) when compared to the baseline values. It was also significantly higher 4 hours after PCI and then returned to the baseline values after 24 hours and started to decrease after 48 hours. When contrast nephropathy was defined as an increase in serum creatinine by >25% of the baseline level 48 hours after PCI, the prevalence of CIN was 10%. Patients with CIN received significantly more contrast agent (*P* < 0.05), but durations of PCI were similar. Midkine was significantly higher 2, 4, and 8 hours after PCI in patients with CIN. Since the “window of opportunity” is narrow in CIAKI and time is limited to introduce proper treatment after initiating insult, particularly when patients are discharged within 24 hours after the procedure, midkine needs to be investigated as a potential early marker for renal ischemia and/or nephrotoxicity.

## 1. Introduction

Midkine (MK; gene name, Mdk), a heparin-binding growth factor, regulates cell growth, cell survival, migration and antiapoptotic activity in nephrogenesis, and development [1]. In addition, MK is involved in inflammation, as revealed by in vivo studies on arterial restenosis [2], rheumatoid arthritis, ischemic renal injury [3], and cisplatin-induced tubulointerstitial [1], and diabetic nephropathy [4]. In the kidney, MK is expressed in both proximal tubular cells and distal tubular epithelial cells [3] and to a lesser extent in endothelial cells [4] and is induced by oxidative stress through the activation of hypoxia-inducible factor-1a [3]. The pathophysiological roles of MK are diverse, ranging from the occurrence of acute kidney injury (AKI) to progression of chronic kidney disease, often accompanied by renal ischemia and diabetic nephropathy [5, 6]. AKI develops as an important and potentially devastating complication with severe hypertension, and its incidence has been reported to vary from 5% in hospitalized patients to 30–50% in intensive care units in the past two decades [7, 8]. Renal ischemia, one of the major causes of AKI, has been intensely linked with damage in various organs through the interorgan interactions involving the kidney by several chemokines [9].

Since interventional cardiologists are being asked more frequently to perform percutaneous coronary intervention (PCI) on increasing numbers of patients, contrast nephropathy (CIN), a form of acute kidney injury, is a potentially serious complication [10, 11]. Peak creatinine typically occurs 3 to 5 days after contrast administration and returned to baseline (or a new baseline) in 1 to 3 weeks [10], when patients are discharged from the hospital. Unfortunately, creatinine is an unreliable indicator during acute changes in kidney function [12]. In current research, several candidates have been proposed as early detection markers of acute renal failure. Some estimate glomerular filtration rate (cystatin C); some reflect renal injury (actin, kidney injury molecule-1, etc.), and others show inflammation associated with acute renal failure (interleukins 6, 8, and 18) [13–15]. In our previous study we reported a rise in serum NGAL after 2 and 4 hours, and a rise in urinary NGAL after 4 and 12 hours after PCI [16].

Taking all these data into consideration, we designed a prospective trial to test the hypothesis whether midkine could represent an early biomarker of contrast nephropathy in patients with normal serum creatinine. We also investigate the eventual relation with the type of coronary intervention and prevalence of contrast nephropathy in this population.

## 2. Methods

The study was performed on 89 consecutive patients undergoing elective PCI due to stable angina (II/III CCS class). We excluded patients with preexisting chronic kidney disease (more than 1.5 mg/dL in males and less than 1.2 mg/dL in females) and chose population with normal serum creatinine, since in patients with impaired renal function we are aware of CIN. None of the patients investigated had received nephrotoxic drugs at least 1 week before and during the study period. All the patients were informed about the aim of the study and gave their consent; the protocol was approved by the local Ethics Committee. All the clinical and biochemical data are given in Table 1. In all the patients 24 h before PCI all the nephrotoxic drugs (NSAIDs, diuretics, and biguanide derivatives in diabetic patients) were withdrawn and ACE inhibitors were either withdrawn (when blood pressure permitted) or halved 24 hours before the procedure. All the patients admitted to the department of invasive cardiology were recommended to drink about 2 liters of still water within 24 hours periprocedurally, ideally 1 liter before PCI and 1 liter within 2 hours after as reported previously [17].

Low-osmolal contrast agent was used in all patients (iodixanol). All the patients were on statins and ACEi. Serum midkine, serum and urinary NGAL, and serum cystatin C were evaluated before and after 2, 4, 8, 24, and 48 hours after PCI. Serum creatinine and urea were assessed before PCI and 24 and 48 hours after the procedure. Hemoglobin, hematocrit, uric acid, cholesterol, HDL, triglycerides, fasting glucose, ejection fraction, and LVIDd (left ventricular internal end-diastolic dimension) were studied at admission. Serum creatinine was measured by the standard laboratory method (Jaffe) in the one central laboratory at the University Hospital. We assessed kidney function according to the simplified MDRD [18], Cockcroft-Gault [19], and CKD-EPI [20] formulae. NGAL was evaluated using commercially available ELISA from ANTIBODYSHOP (Gentofte, Denmark). All tests were performed according to manufactures' instructions by the same person. Serum midkine was measured using commercially available kits from Biovendor, Austria. Data given were analyzed using Statistica 10.0. ANOVA or Kruskal-Wallis ANOVA and Kruskal-Wallis ANOVA for repeated measurements were used in statistical analysis with *P* < 0.05 considered statistically significant, when appropriate.

## 3. Results

Clinical and biochemical characteristics of the population studies are presented in Table 1. In the whole group studied we found a significant rise in serum midkine as early as after 2 hours (*P* < 0.01) when compared to the baseline values. It was also significantly higher 4 hours after PCI (*P* < 0.05) then returned to the baseline values after 24 hours and started to decrease after 48 hours (Table 2). Serum NGAL increased after 2, 4, and 8 hours and in urinary NGAL after 4, 8, and 24 hours after PCI. We found a significant rise in serum NGAL after 2, 4, and 8 hours and in urinary NGAL after 4, 8, and 24 hours after PCI. Serum cystatin C increased significantly after 8 hours, reaching peak 24 hours after PCI, and then decreased after 48 hours. When contrast nephropathy was defined as an increase in serum creatinine by >25% of the baseline level 48 hours after PCI, the prevalence of CIN was 10%. Patients with CIN received significantly more contrast agent (206.76 ± 108.76 mL versus 168.43 ± 69.43 mL, *P* < 0.05), but duration of PCI was similar.

**Figure 1:**
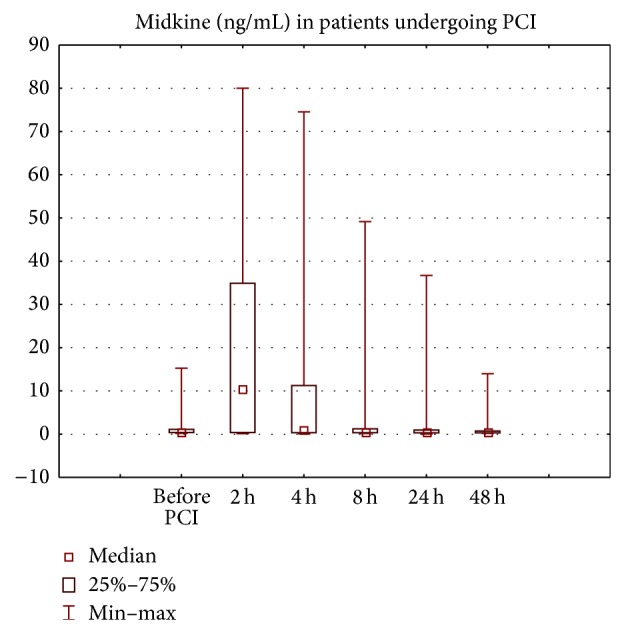
Midkine in patients with CIN (after 2 hours *P* < 0.001, after 4 hours *P* < 0.01, and after 8 hours *P* < 0.05 versus baseline).

Midkine was significantly higher 2, 4, and 8 hours after PCI in patients with CIN (Figure 1), while NGAL levels were significantly higher in patients with CIN starting 2 hours after PCI (serum NGAL) or 4 hours (urinary NGAL). Cystatin C was higher only 8 and 24 hours after PCI in patients with CIN (data not shown).

Midkine correlated in univariate correlation with time of PCI (*r* = 0.43, *P* < 0.01), amount of contrast agent (*r* = 0.26, *P* < 0.05), systolic blood pressure (*r* = 0.30, *P* < 0.05), presence of diabetes (*r* = 0.25, *P* < 0.05), serum creatinine (*r* = 0.29, *P* < 0.05), and urea (*r* = 0.25, *P* < 0.05). In addition, midkine 2 hours after PCI correlated with serum NGAL at the same time point (*r* = 0.27, *P* < 0.05), 24 hours after PCI with NGAL at the same time point (*r* = 0.28, *P* < 0.05), and 48 hours after PCI with NGAL at the same time point (*r* = 0.70, *P* < 0.001).

## 4. Discussion

We have found that midkine is a sensitive marker of renal injury after contrast administration in low-risk patients undergoing percutaneous coronary interventions. We found a significant rise in serum midkine as early as after 2 hours (*P* < 0.001) when compared to the baseline values. It was also significantly higher 4 hours after PCI then returned to the baseline values after 24 hours and started to decrease after 48 hours. Midkine is multifunctional heparin-binding growth factor with various biological roles including inflammation [20]. Role of midkine in inflammatory process such as ischemic kidney injury was studied by Sato et al. [3]. Contrast nephropathy is a generally reversible form of acute kidney injury (AKI) that occurs soon after the administration of radiocontrast media [10] Pathophysiological processes thought to contribute to the development of CIAKI include renal vasoconstriction leading to medullary ischemia, direct tubular cytotoxicity of contrast, and the generation of reactive oxygen species which contribute to cell damage [11].

During renal ischemia, depletion of energy in renal epithelial cells occurs and affects various beneficial and deleterious life systems. It is responsible for disruption of the cytoskeleton and cell polarity and cell death, or it may indirectly induce chemotaxis through the activation of various kinds of cells such as endothelial cells and leukocytes. In addition, necrosis and autophagy occur after ischemic reperfusion injury. Midkine promotes the migration of neutrophils and macrophages but is expressed at very low levels in proximal tubules. However, after ischemic reperfusion midkine is immediately induced in the proximal tubules. This leads to the upregulation of macrophage inflammatory protein-2 for neutrophils and monocyte chemotactic protein- (MCP-) 1 for macrophages [3, 21]. Eventually, infiltrated inflammatory cells cause severe tubulointerstitial injury. Midkine inhibition can prevent the migration of inflammatory cells to the injured epithelial layer, reducing the severity of renal damage. It was showed by Sato et al. [3] in midkine-deficient mice. In these animals migration of inflammatory cells to the epithelial layer was diminished and thus renal injury was less severe. Their results indicate that midkine enhances migration of inflammatory cells on ischemic injury of the kidney directly and also through induction of chemokines and contributes to the augmentation of ischemic tissue damage.

Current kidney injury biomarkers, especially creatinine and protein in urine, are inadequate and other conventional biomarkers as urinary casts and fractional sodium excretion have been found insensitive and nonspecific for the early detection of AKI. Similarly other traditional biomarkers detected in urine such as filtered low-molecular weight proteins, tubular proteins, or enzymes have also suffered from lack of specificity and standardized assays. Thus, different urinary and serum proteins have been intensively investigated as possible biomarkers for the early diagnosis of AKI. As tubular injury, due to direct cytotoxic effects or in association with the generation of oxygen free radicals, contributes to contrast nephropathy, we hypothesized that midkine may serve as a potential marker of acute kidney injury.

In addition we took into account the fact that midkine is also expressed at a very low level [22] in veins and arteries under healthy conditions. We studied patients with coronary artery disease. Atherosclerosis is a chronic inflammatory disease of large- and medium-sized arteries characterized by endothelial dysfunction and subsequent plaque formation of the vascular wall. During PCI an injury to the endothelium may lead to increased midkine expression, as it was shown in different animal models [2, 23]. It was reported that macrophages infiltrating the injured vascular wall after stenting have been found to be a major source of midkine, whereas freshly isolated monocytes did not express MK [22]. From the clinical perspective, during PCI vessel injury is followed by an inflammatory response causing neointima formation which may lead to restenosis after intervention. In experimental animals, neointima formation was almost completely absent in midkine-deficient mice relative to control mice [2]. However, intra-arterial administration of recombinant MK restored neointima formation [2].

Vascular restenosis occurred at a high rate in patients who receive vascular reconstruction using procedures such as ballooning, stenting, and grafting, performed in the invasive cardiology or cardiac surgery units. This was the rationale to introduce dual antiplatelet therapy to prevent such complications. Early restenosis was more prevalent with bare metal stents, while late restenosis (after 12 months) was more prevalent with drug-eluting stents. Target lesion revascularization ranged from 5% to 35%, stent thrombosis from 0% to 9%, and mortality rate from 0% to 8% [24]. The formation of neointima is the basic lesion, and midkine expression is induced in this lesion and midkine promotes migration of inflammatory cells and smooth muscle cells, and this activity is thought to cause neointima formation. Thus the rise in serum midkine could be attributed to both tubular injury and endothelium injury in coronary arteries. However, the proportions are unknown. Similar situations were reported for NGAL; it was considered as renal troponin, but it was also present in the atherosclerotic plaques [25]. We might speculate as in the case of NGAL that the substantial amount of midkine comes from kidney not from coronary artery endothelium. However, further studies are needed to prove or disprove this hypothesis.

As we stressed in the recent review, kidney-specific biomarkers have seen very limited clinical application [26], despite availability for clinical use in several regions worldwide; however, they appeared earlier than serum creatinine. In contrast nephropathy the “window of opportunity” is narrow and time is limited to introduce proper treatment after initiating insult, particularly when patients are discharged within 24 hours after PCI. In addition, lack of biomarkers delays our ability to institute effective therapy. However, we are far advanced in the search for “the troponin of the kidney”; however, we should be aware that acceptance of troponin as a cardiac marker was also a long process and now with these new cardiac biomarkers we change the paradigms shift. Time for kidney troponin(s) is to come, the sooner, the better for our patients. Whether midkine might help us to diagnose contrast nephropathy, time will bring us an answer.

## Figures and Tables

**Table 1:** Basal clinical characteristics in patients undergoing elective PCI.

Parameters	Number and percentage
Age (years)	63.05 ± 12.06
BMI (kg/m^2^)	24.87 ± 6.89
SBP (mm Hg)	136.13 ± 32.02
DBP (mm Hg)	91.09 ± 17.87
Hemoglobin (g·dL)	15.03 ± 2.67
Hematocrit (%)	41.23 ± 7.65
HbA1c (%)	5.02 ± 2.07
Albumin (g/L)	3.87 ± 0.67
Urea (mg/dL)	44.23 ± 22.05
Creatinine (mg/dL)	1.05 ± 0.36
Cockcroft-Gault formula (mL/min)	64.76 ± 24.87
MDRD equation (mL/min/1.72 m^2^)	81.89 ± 27.65
CKD-EPI (mL/min/1.72 m^2^)	83.76 ± 29.76
Cholesterol (mg/dL)	172.89 ± 54.07
HDL (mg/dL)	44.07 ± 14.34
Triglycerides (mg/dL)	212.65 ± 67.82
Uric acid (mg/dL)	5.32 ± 1.73
Fasting glucose (mg/dL)	109.76 ± 59.52
Ejection fraction (%)	44.54 ± 18.24
LVIDd (left ventricular internal end-diastolic dimension) (mm)	4.29 ± 1.94
Duration of PCI (mins)	56.29 ± 24.54
Contrast volume (mL)	168.92 ± 85.45
Hypertension	80/89
Diabetes	37/89

**Table 2:** Kidney function assessed by serum and urinary NGAL, serum and urinary creatinine, and cystatin C in 89 patients undergoing PCI.

	Before coronary angiography	Two hours	Four hours	Eight hours	24 hours	48 hours
Serum NGAL (ng/mL)	103.26 ± 63.37	125.82 ± 67.34^*^	139.70 ± 87.65^*^	119.76 ± 71.5	117.34 ± 66.65	98.56 ± 46.89
Urinary NGAL (ng/mL)	0 (0.0; 9.1)	1.2 (1.5; 14.0)	3.8 (4.8; 20.6)^*^	4.1 (5.8; 32.3)^*^	1.7 (2.7; 18.3)	0 (0; 8; 4)
Cystatin C (mg/L)	1.55 ± 1.06	1.69 ± 1.07	2.09 ± 1.17	1.99 ± 1.26	2.59 ± 1.05^**^	1.80 ± 1.07
Creatinine (mg/dL)	1.05 ± 0.36	ND	ND	ND	1.25 ± 0.49	1.18 ± 0.38
Midkine (ng/mL)	0.50 (3.81; 1.10)	2.46 (0.39; 10.45)^**^	0.98 (0.35; 1.12)^*^	0.51 (0.31; 1.10)	0.48 (0.30; 0.95)	0.45 (0.31; 0.72)

Data given are means ± SD or medians and interquartile ranges.

^*^ *P* < 0.05, ^**^ *P* < 0.01 versus baseline.
